# Supplementary material for: Modulating the Viscoelastic Properties of Covalently Crosslinked Protein Hydrogels
Source: Gels. 2023 Jun 12;9(6):481. doi: 10.3390/gels9060481 (PMC10298573; doi:10.3390/gels9060481)
Supplement: Supplementary file 1 [file gels-09-00481-s001.zip › gels-2356642-supplementary.pdf]

Supplementary material

Modulating the viscoelastic properties of covalently crosslinked protein hydrogels

Boni and Regan

Contents:

Supplementary figures:

- Supplementary figure S1: swelling properties of the protein hydrogels.
- Supplementary figure S2: SasGlong and SC3
- Supplementary figure S3: SasG and SC4
- Supplementary figure S4: SasGlong and SC4
- Supplementary figure S5: Frequency sweep of ST-SasG-ST:SC3 at progressively increasing total protein concentrations, with ST:SC = 1.
- Supplementary figure S6: Frequency sweep of ST-SasGlong-ST:SC3 at progressively increasing total protein concentrations, with ST:SC = 1.
- Supplementary figure S7: Frequency sweep of ST-SasG-ST:SC4 at progressively increasing total protein concentrations, with ST:SC = 1.
- Supplementary figure S8: Frequency sweep of ST-SasGlong-ST:SC4 at progressively increasing total protein concentrations, with ST:SC = 1.
- Microrheology methods
- Supplementary Figure S9. Gelation kinetics of ST-SasG-ST and SC3 1.3:1 and 2.6:1.
- Supplementary Figure S10. Gelation kinetics of ST-SasGlong-ST and SC3 1.3:1 and 2:1.

1. Plasmids used in this study: DNA and protein sequences
2. Strains used in this study
3. Raw data provided as a separate excel file.

Supplementary Figure S1.

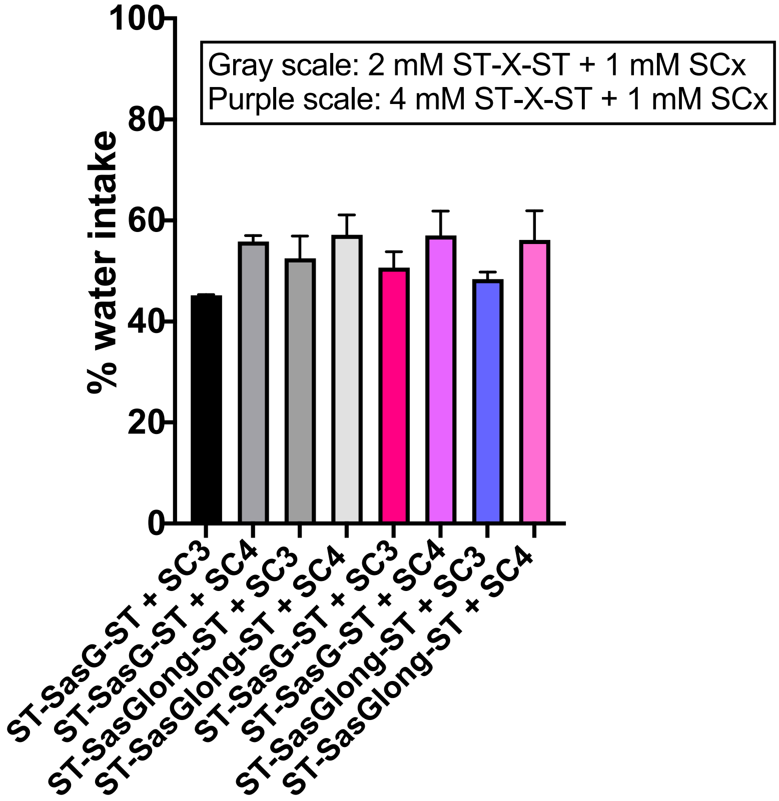

Supplementary figure S1: Swelling properties of the protein hydrogels at different ST:SC ratios.

Supplementary figure S2.

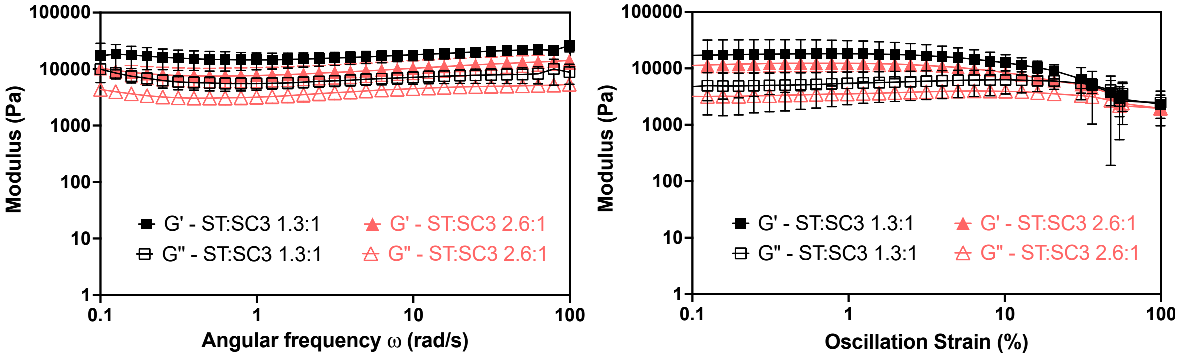

Supplementary Figure S2: Black: ST-SasGlong-ST:SC3 1.3:1; salmon: ST-SasGlong-ST:SC3 2.6:1. Combinations of ST-SasGlong-ST and SC3 exhibited classic gel like behaviour, with G'>G" and G' = 10,000, as expected from a hydrogel with critical yield stress at ~10% strain (G' <G").

Supplementary figure S3.

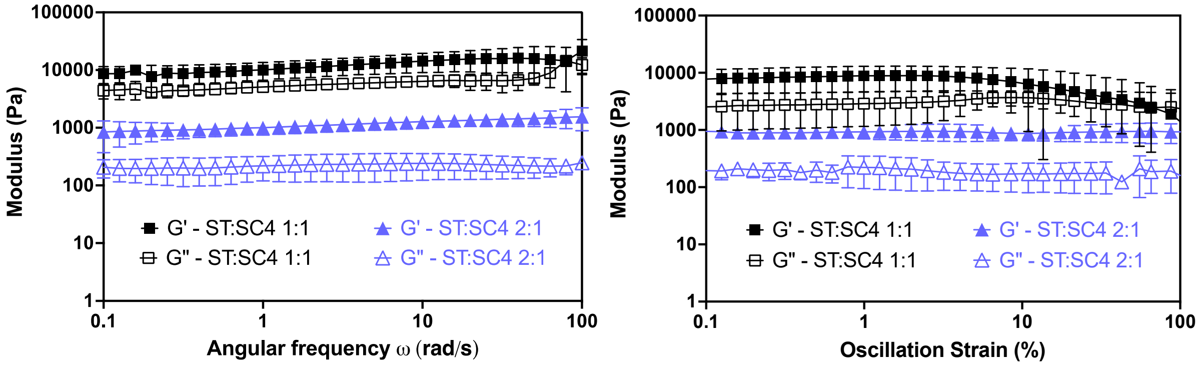

Supplementary figure S3. Black: ST-SasG-ST:SC4 1:1 exhibited classic gel like behaviour, with G'>G" and G' = 10,000, as expected from a hydrogel, and critical yield stress at 10% strain (G' <G"). Purple: ST-SasG-ST:SC4 2:1 showed a 10-fold reduction in G' to 1,000 Pa, and a greater resistance to deformation, up to 100% strain.

Supplementary figure S4.

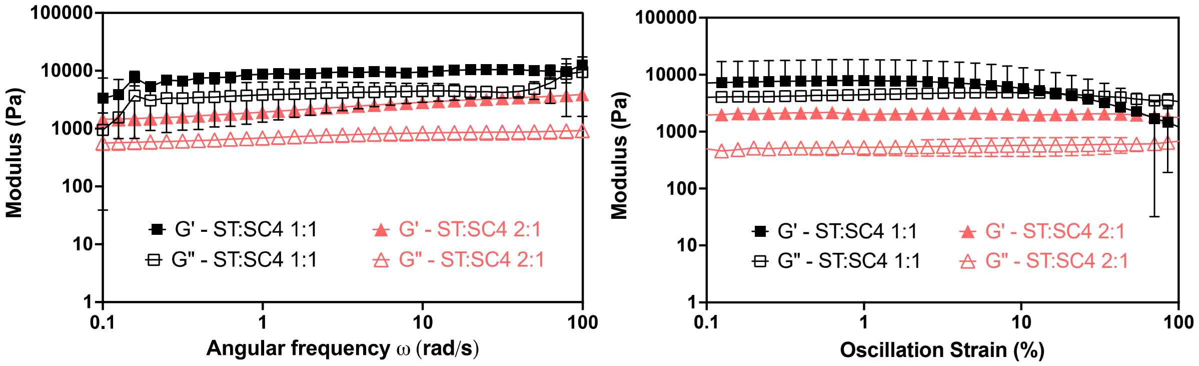

Supplementary figure S4. Black: ST-SasGlong-ST:SC4 1:1 exhibited classic gel like behaviour, with G'>G" and G' = 10,000, as expected from a hydrogel, and critical yield stress at 10% strain (G' <G"). Salmon: ST-SasGlong-ST:SC4 2:1 showed a 10-fold reduction in G' to 1,000 Pa, and a greater resistance to deformation, up to 100% strain.

Supplementary figure S5.

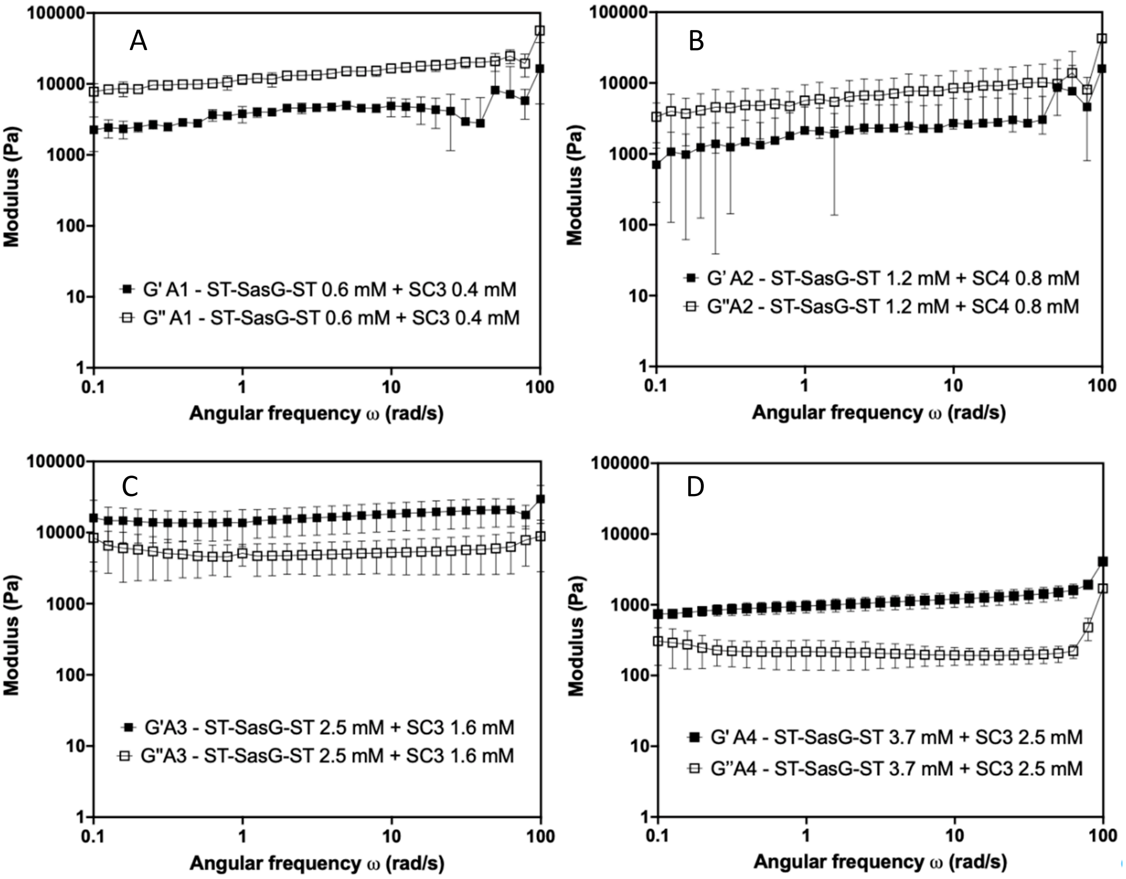

Supplementary figure S5. Frequency sweeps of ST-SasG-ST:SC3 at progressively increasing total protein concentrations with ST:SC = 1. A) ST:SC = 0.6:0.4 mM, G'< G", indicative of viscous liquid. B) ST:SC = 1.2: 0.8 mM, G'<G" indicative of viscous liquid. C) ST:SC = 2.5:1.6 mM, G'>G" and G'= 10,000 Pa, indicative gel like behaviour. D) ST:SC = 3.7:2.5 mM, G'>G" and G' = 1,000 Pa, indicative of gel like behaviour.

Supplementary figure S6.

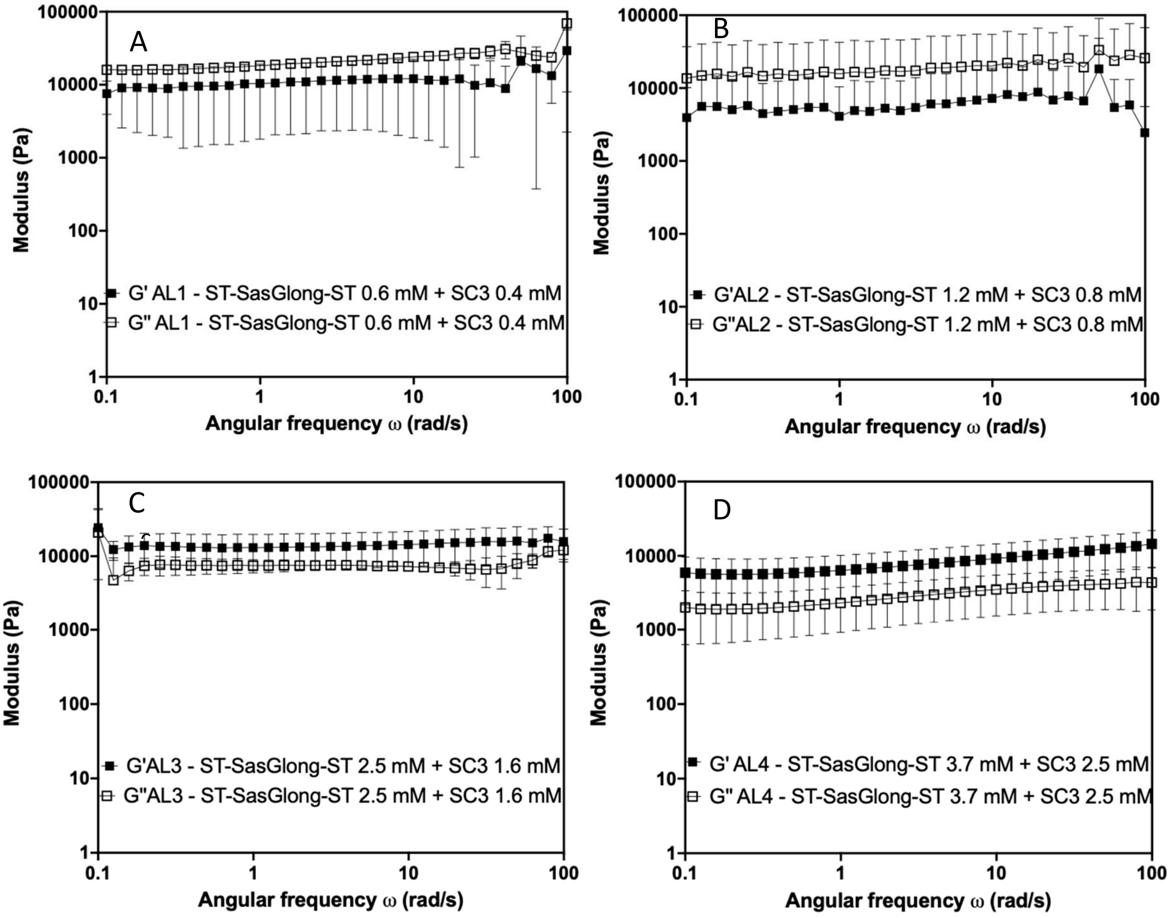

Supplementary figure S6. Frequency sweeps of ST-SasGlong-ST:SC3 at progressively increasing total protein concentrations with ST:SC = 1. A) ST:SC = 0.6:0.4 mM, G'< G", indicative of viscous liquid. B) ST:SC = 1.2: 0.8 mM, G'<G" indicative of viscous liquid. C) ST:SC = 2.5:1.6 mM, G'>G" and G'= 10,000 Pa, indicative gel like behaviour. D) ST:SC = 3.7:2.5 mM, G'>G" and G' = 1,000 Pa, indicative of gel like behaviour.

Supplementary figure S7.

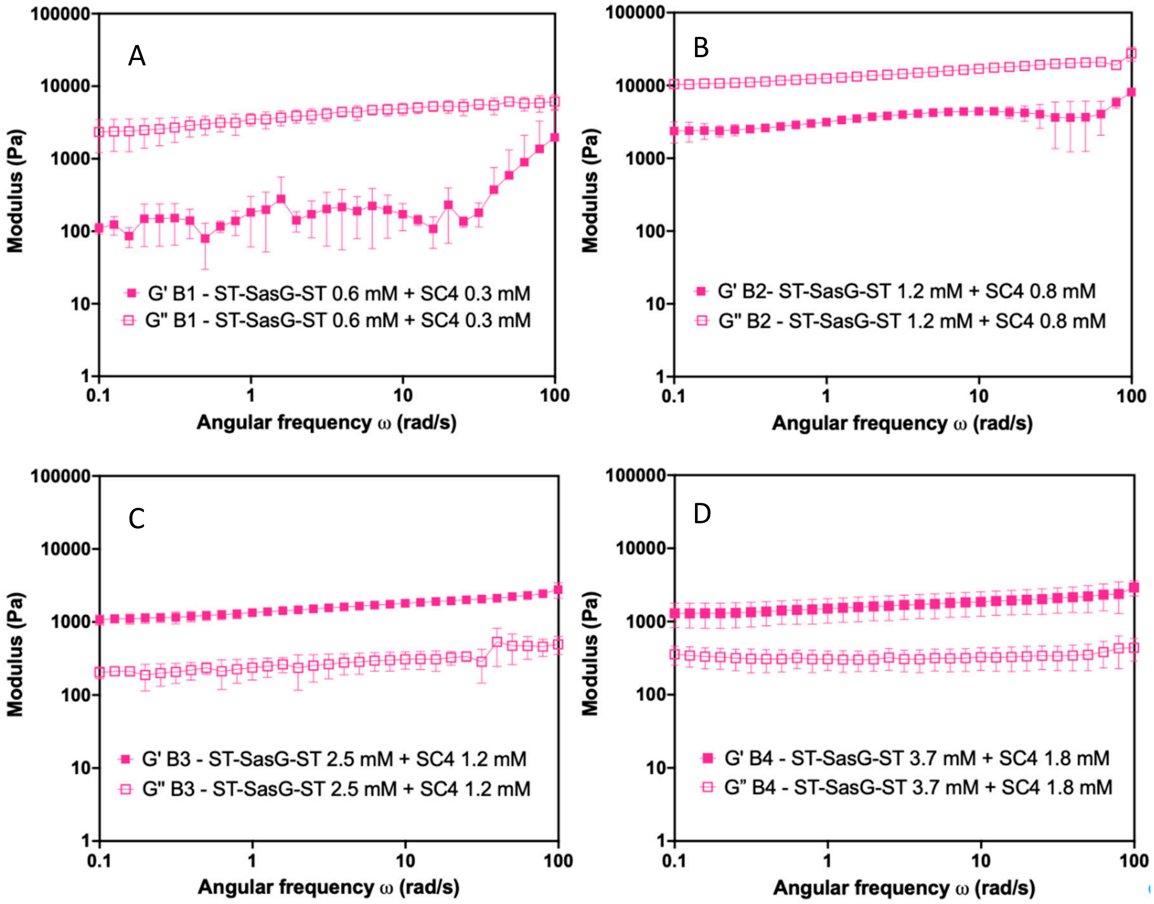

Supplementary figure S7. Frequency sweeps of ST-SasG-ST:SC4 at progressively increasing total protein concentrations with ST:SC = 1. A) ST:SC = 0.6:0.3 mM, G'< G", indicative of viscous liquid. B) ST:SC = 1.2:0.8 mM, G'<G" indicative of viscous liquid. C) ST:SC = 2.5:1.2 mM, G'>G" and G'= 10,000 Pa, indicative gel like behaviour. D) ST:SC = 3.7:1.8 mM, G'>G" and G' = 1,000 Pa, indicative of gel like behaviour.

Supplementary figure S8.

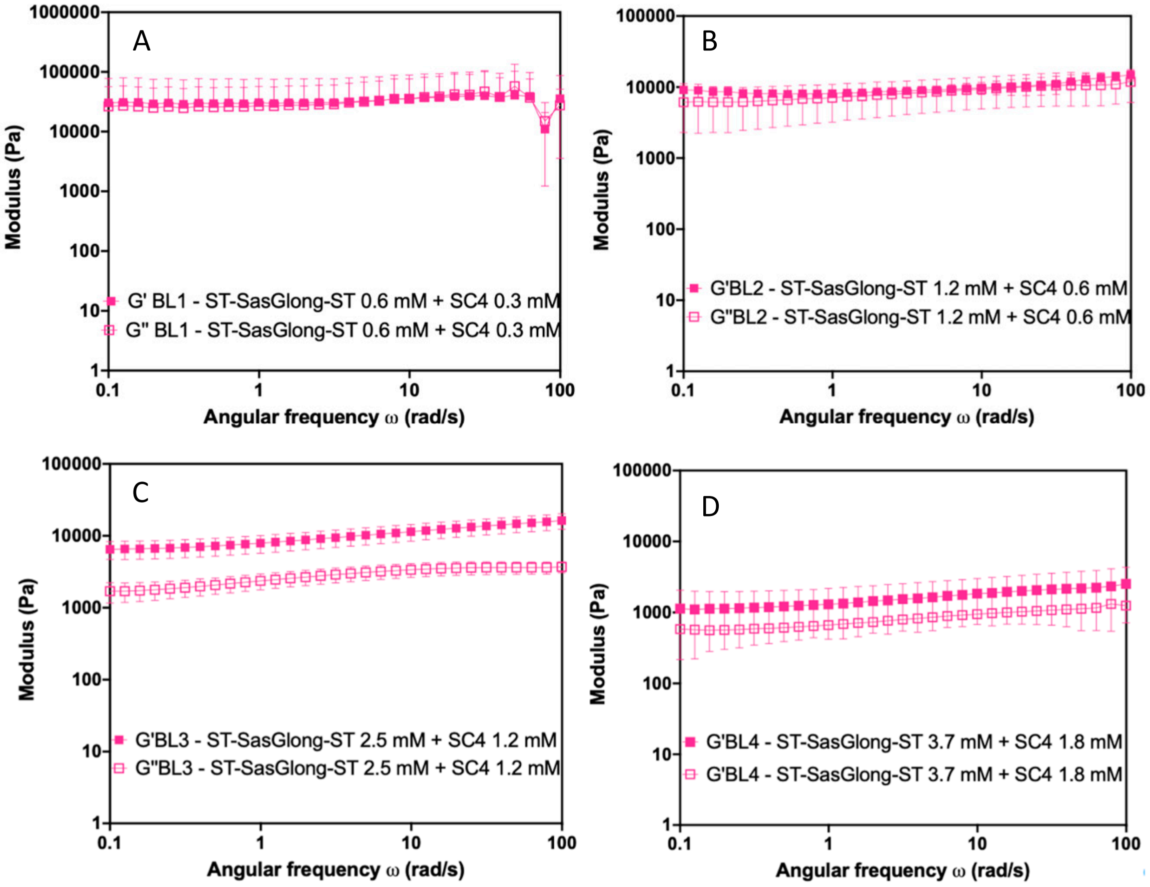

Supplementary figure S8. Frequency sweeps of ST-SasGlong-ST:SC4 at progressively increasing total protein concentrations with ST:SC = 1. A) ST:SC = 0.6:0.3 mM, G'< G", indicative of viscous liquid. B) ST:SC = 1.2:0.8 mM, G'<G" indicative of viscous liquid. C) ST:SC = 2.5:1.2 mM, G'>G" and G'= 10,000 Pa, indicative gel like behaviour. D) ST:SC = 3.7:1.8 mM, G'>G" and G' = 1,000 Pa, indicative of gel like behaviour.

Microrheology methods

Data collection started immediately after spontaneous gelation was initiated. The embedded tracer particles were imaged in brightfield at a magnification of 60x using a Nikon Eclipse Ti inverted microscope (numerical aperture 0.7, 60 x air immersion Extra Long Working Distance objective, Nikon, Japan). The Extra Long Working Distance objective allowed for imaging *in vitro* through the coverslip without introduction of aberrations and subsequent deterioration of the image quality. The movement of roughly 50 in-frame particles was recorded for a total of 6000 frames per minute (100 frames per second, fps) using a CMOS high speed camera (ORCA – Flash 4.0 V3, Hamamatsu) and the Micromanager V1.4.19 software[1]. Static errors in determining particle centroid due to intrinsic variations in the experimental set up were minimised by spreading the tracer beads over a sufficient number of pixels to represent the particle's brightness distribution reasonably[2]. This was achieved by keeping the illumination levels near the maximum allowed by detector saturation. Dynamic errors due to mismatches between the movement of the particles and image acquisition speed were minimised by selecting a short exposure time (σ = 1000 μs) [3]. The samples were imaged in real time for a minimum of 45 minutes and a maximum of 110 minutes. Particle tracking was performed using a Phyton script based on the weighted centroid method developed by Crocker and Grier [4] and the ensemble-averaged mean square displacement $\langle \Delta r^2(\tau) \rangle$ was calculated.

Supplementary Figure S9.

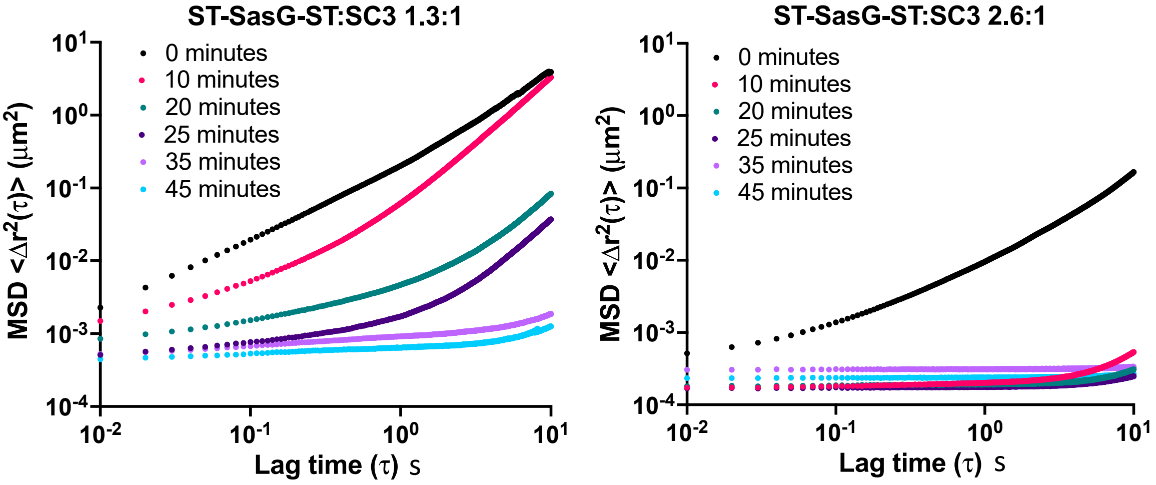

Supplementary Figure S9. Gelation kinetics of ST-SasG-ST and SC3 1.3:1 and 2.6:1. Immediately after mixing of ST and SC, t = 0, both networks showed liquid-like behaviour and free diffusion of the microparticles in the system ($\langle \Delta r^2(\tau) \rangle \approx \tau$). As the time post mixing increased, the behaviour of both systems changed. The ST:SC 1.2:1 showed a progressive decrease in MSD and dependence on lag time until the system reached a plateau $\alpha = 0$, indicative of full gelation at ~ 45 minutes. The ST:SC 2.6:1 system showed a fast decline of MSD, that approached a constant value $\alpha = 0$ as early as 10 minutes.

Supplementary figure S10.

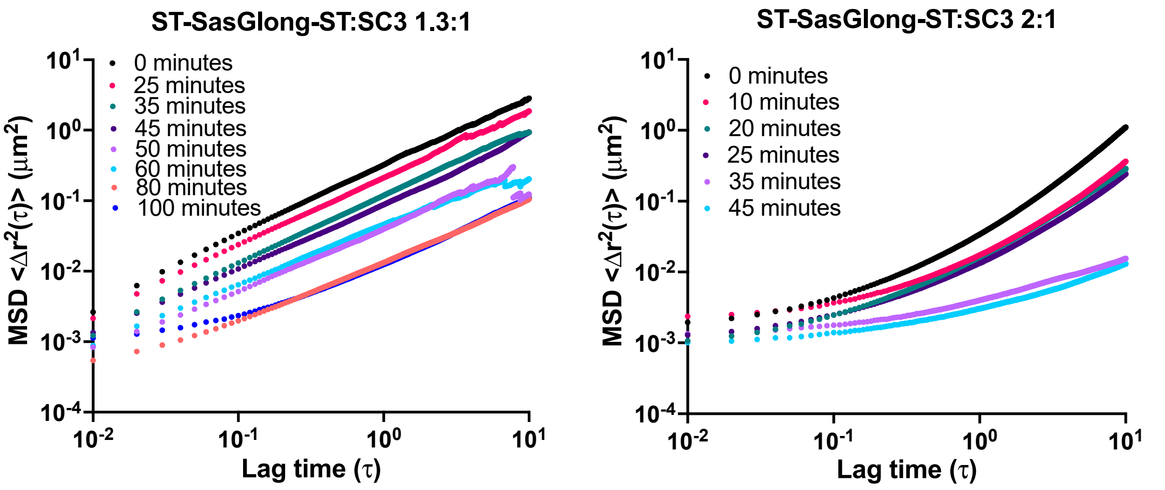

Supplementary figure S10. Gelation kinetics of ST-SasGlong-ST and SC3 1.3:1 and 2:1. Immediately after mixing of ST and SC, t = 0, both networks showed liquid-like behaviour and free diffusion of the microparticles in the system ($<\Delta r^2(\tau)> \approx \tau$). As the time post mixing increased, the behaviour of both systems changed. The ST:SC 1.3:1 showed a small decrease in MSD and dependence on lag time. The system never reached the plateau $\alpha = 0$, indicative of the presence of a viscous component in the hydrogel. The ST:SC 2:1 system showed a faster decline of MSD, but similarly never reached the constant value $\alpha = 0$.

Table S1. Molar concentration of SC3 and SC4 in combination with the ST crosslinkers SasG or SasGlong.

SC3 [mM]	SC4 [mM]	ST-crosslinker-ST [mM]
0.4	-	0.6
0.8	-	1.2
1.6	-	2.5
2.5	-	3.7
-	0.3	0.6
-	0.6	1.2
-	1.2	2.5
-	1.8	3.7

1. **Plasmids used in this study**

Each plasmid is detailed in the following pages, with both DNA coding sequence and protein amino acid sequence annotated with colour for clarity. All proteins are expressed via the pTrc promoter. Plasmids are used to express the following proteins:

1. SpyTag-SasG-SpyTag
2. SpyTag-SasGlong-SpyTag
3. SpyCatcher – GSx3 array
4. SpyCatcher – GSx4 array

SpyTag-SasG-SpyTag

**Name**	ST-SasG-ST	**Source**	5
**Resistance**	Ampicillin	**Total plasmid size (bp)**	5403
**Parent Vector**	pPROEX HTa	**Seq Primers**	M13_pUC +pBAD
**Benchling link**	https://benchling.com/s/seq-eyHiB1hKBXfQfuZJAppg?m=slm-pDPyP77iqjd4gYqZlcsj		

Description

SasG protein flanked by one SpyTag at the N and C termini. Cleavable N terminal His tag.

Shading Key

His-TEV-ST-SasG-ST

DNA

ATGTCGTACTACCATCACCATCACCATCACGATTACGATATCCCAACGACCGAA
AACCTGTATTTTCAGGGCGCCATGGGATCCGCGCATATTGTAATGGTGGATGCT
TACAAACCCACGAAACATATGGCACCGAAAACCATCACCGAACTGGAAAAGAAG
GTGGAAGAAATCCCGTTCAAGAAAGAACGCAAATTCAACCCGGACCTGGCACC
GGGTACCGAAAAAGTGACGCGTGAAGGCCAGAAAGGTGAAAAAACCATTACCA
CGCCGACGCTGAAAAACCCGCTGACCGGCGTTATTATCAGCAAAGGTGAACCG
AAAGAAGAAATTACCAAAGATCCGATCAATGAACTGACCGAATATGGCCCGGAA
ACGATTGCGCCGGGTCATCGTGATGAATTTGACCCGAAACTGCCGACCGGCGA
AAAAGAAGAAGTGCCGGGCAAACCGGGTATCAAAAATCCGGAAACCGGCGATG
TGGTTCGCCCGCCGGTCGACAGTGTGACGAAATACGGCCCGGTTAAGGGTGAT
TCCATTGTCGAAAAAGAAGAAATCCCGTTTGAAAAAGAACGTAAATTTAATCCGG
ACCTGGCGCCGGGTACCGAAAAAGTTACGCGCGAAGGCCAAAAAGGTGAAAAA
ACCATCACGACGCCGACCCTGAAAAATCCGCTGACCGGCGAAATTATCAGCAA
GGGTGAATCTAAAGAAGAAATCACGAAAGACCCGATCAACGAACTGACCGAATA
CGGCCCGGAAACCATTGCCCCCGGGGCGCATATTGTAATGGTGGATGCTTACA
AACCCACGAAATGA

Expression Product Sequence

MSYYHHHHHHDYDIPTTENLYFQGAMGSAHIVMVDAYKPTKHMAPKTITELEKKVE
EIPFKKERKFNPDLAPGTEKVTREGQKGEKTITTPTLKNPLTGVIISKGEPKEEITKDP
INELTEYGPETIAPGHRDEFDPKLPTGEKEEVPGKPGIKNPETGDVVRPPVDSVTKY
GPVKGDSIVEKEEIPFEKERKFNPDLAPGTEKVTREGQKGEKTITTPTLKNPLTGEII
SKGESKEEITKDPINELTEYGPETIAPGAHIVMVDAYKPTK*

SpyTag-SasGlong-SpyTag

Name	ST-SasGlong-ST	Source	5
**Resistance**	Ampicillin	**Total plasmid size (bp)**	6939
**Parent Vector**	pPROEX HTa	**Seq Primers**	M13_pUC +pBAD
**Benchling link**	https://benchling.com/s/seq-S4rvBMTg4MwD94VlV7tx?m=slm-zfk0j2Ogdyo5kKHyvMHT		

Description
SasGlong protein (GEG+3x(EG)) flanked by one SpyTag at the N and C termini. Cleavable N terminal His tag.

Shading Key
His-TEV-ST-SasGlong-ST

DNA
ATGTCGTACTACCATCACCATCACCATCACGATTACGATATCCCAACGACCGAA
AACCTGTATTTTCAGGGCGCCATGGGATCCGCGCATATTGTAATGGTGGATGCT
TACAAACCCACGAAACATATGGCACCGAAAACCATCACCGAACTGGAAAAGAAG
GTGGAAGAAATCCCGTTCAAGAAAGAACGCAAATTCAACCCGGACCTGGCACC
GGGTACCGAAAAAGTGACGCGTGAAGGCCAGAAAGGTGAAAAAACCATTACCA
CGCCGACGCTGAAAAACCCGCTGACCGGCGTTATTATCAGCAAAGGTGAACCG
AAAGAAGAAATTACCAAAGATCCGATCAATGAACTGACCGAATATGGCCCGGAA
ACGATTGCGCCGGGTCATCGTGATGAATTTGACCCGAAACTGCCGACCGGCGA
AAAAGAAGAAGTGCCGGGCAAACCGGGTATCAAAAATCCGGAAACCGGCGATG
TGGTTCGCCCGCCGGTCGACAGTGTGACGAAATACGGCCCGGTTAAGGGTGAT
TCCATTGTCGAAAAAGAAGAAATCCCGTTTGAAAAAGAACGTAAATTTAATCCGG
ACCTGGCGCCGGGTACCGAAAAAGTTACGCGCGAAGGCCAAAAAGGTGAAAAA
ACCATCACGACGCCGACCCTGAAAAATCCGCTGACCGGCGAAATTATCAGCAA
GGGTGAATCTAAAGAAGAAATCACGAAAGACCCGATCAACGAACTGACCGAATA
CGGCCCGGAAACCATTGCCCCCGGTCATCGTGATGAATTTGACCCGAAACTGC
CGACCGGCGAAAAAGAAGAAGTGCCGGGCAAACCGGGTATCAAAAATCCGGAA
ACCGGCGATGTGGTTCGCCCGCCGGTCGACAGTGTGACGAAATACGGCCCGG
TTAAGGGTGATTCCATTGTCGAAAAAGAAGAAATCCCGTTTGAAAAAGAACGTA
AATTTAATCCGGACCTGGCGCCGGGTACCGAAAAAGTTACGCGCGAAGGCCAA
AAAGGTGAAAAAACCATCACGACGCCGACCCTGAAAAATCCGCTGACCGGCGA
AATTATCAGCAAGGGTGAATCTAAAGAAGAAATCACGAAAGACCCGATCAACGA
ACTGACCGAATACGGCCCGGAAACCATTGCCCCCGGTCATCGTGATGAATTTG
ACCCGAAACTGCCGACCGGCGAAAAAGAAGAAGTGCCGGGCAAACCGGGTAT
CAAAAATCCGGAAACCGGCGATGTGGTTCGCCCGCCGGTCGACAGTGTGACG
AAATACGGCCCGGTTAAGGGTGATTCCATTGTCGAAAAAGAAGAAATCCCGTTT
GAAAAAGAACGTAAATTTAATCCGGACCTGGCGCCGGGTACCGAAAAAGTTAC
GCGCGAAGGCCAAAAAGGTGAAAAAACCATCACGACGCCGACCCTGAAAAATC
CGCTGACCGGCGAAATTATCAGCAAGGGTGAATCTAAAGAAGAAATCACGAAA
GACCCGATCAACGAACTGACCGAATACGGCCCGGAAACCATTGCCCCCGGTCA
TCGTGATGAATTTGACCCGAAACTGCCGACCGGCGAAAAAGAAGAAGTGCCGG
GCAAACCGGGTATCAAAAATCCGGAAACCGGCGATGTGGTTCGCCCGCCGGTC
GACAGTGTGACGAAATACGGCCCGGTTAAGGGTGATTCCATTGTCGAAAAAGA
AGAAATCCCGTTTGAAAAAGAACGTAAATTTAATCCGGACCTGGCGCCGGGTAC

CGAAAAAGTTACGCGCGAAGGCCAAAAAGGTGAAAAAACCATCACGACGCCGA
CCCTGAAAAATCCGCTGACCGGCGAAATTATCAGCAAGGGTGAATCTAAAGAA
GAAATCACGAAAGACCCGATCAACGAACTGACCGAATACGGCCCGGAAACCAT
TGCCCCCGGTCATCGTGATGAATTTGACCCGAAACTGCCGACCGGCGAAAAAG
AAGAAGTGCCGGGCAAACCGGGTATCAAAAATCCGGAAACCGGCGATGTGGTT
CGCCCGCCGGTCGACAGTGTGACGAAATACGGCCCGGTTAAGGGTGATTCCAT
TGTCGAAAAAGAAGAAATCCCGTTTGAAAAAGAACGTAAATTTAATCCGGACCT
GGCGCCGGGTACCGAAAAAGTTACGCGCGAAGGCCAAAAAGGTGAAAAAACCA
TCACGACGCCGACCCTGAAAAATCCGCTGACCGGCGAAATTATCAGCAAGGGT
GAATCTAAAGAAGAAATCACGAAAGACCCGATCAACGAACTGACCGAATACGG
CCCGGAAACCATTGCCCCCGGGGCGCATATTGTAATGGTGGATGCTTACAAAC
CCACGAAATGATAA

Expression product sequence:
MSYYHHHHHHDYDIPTTENLYFQGAMGSAHIVMVDAYKPTKHMAPKTITELEKKVE
EIPFKKERKFNPDLAPGTEKVTREGQKGEKTITTPTLKNPLTGVIISKGEPKEEITKDP
INELTEYGPETIAPGHRDEFDPKLPTGEKEEVPGKPGIKNPETGDVVRPPVDSVTKY
GPVKGDSIVEKEEIPFEKERKFNPDLAPGTEKVTREGQKGEKTITTPTLKNPLTGEII
SKGESKEEITKDPINELTEYGPETIAPGHRDEFDPKLPTGEKEEVPGKPGIKNPETG
DVVRPPVDSVTKYGPVKGDSIVEKEEIPFEKERKFNPDLAPGTEKVTREGQKGEKTI
TTPTLKNPLTGEIISKGESKEEITKDPINELTEYGPETIAPGHRDEFDPKLPTGEKEEV
PGKPGIKNPETGDVVRPPVDSVTKYGPVKGDSIVEKEEIPFEKERKFNPDLAPGTEK
VTREGQKGEKTITTPTLKNPLTGEIISKGESKEEITKDPINELTEYGPETIAPGHRDEF
DPKLPTGEKEEVPGKPGIKNPETGDVVRPPVDSVTKYGPVKGDSIVEKEEIPFEKER
KFNPDLAPGTEKVTREGQKGEKTITTPTLKNPLTGEIISKGESKEEITKDPINELTEYG
PETIAPGHRDEFDPKLPTGEKEEVPGKPGIKNPETGDVVRPPVDSVTKYGPVKGDS
IVEKEEIPFEKERKFNPDLAPGTEKVTREGQKGEKTITTPTLKNPLTGEIISKGESKEE
ITKDPINELTEYGPETIAPGAHIVMVDAYKPTK**

SpyCatcher 3

**Name**	SpyCatcher GSx3 array	**Source**	5
**Resistance**	Ampicillin	**Total plasmid size (bp)**	5760
**Parent Vector**	pPROEX HTa	**Seq Primers**	M13_pUC +pBAD
**Benchling link**	https://benchling.com/s/seq-SqSqFQytCEdys80vaAYI?m=slm-0J7C0uJyMB3WBRx5ZwUq		

Description
Three SpyCatcher units linked by GS flexible linker. Cleavable N terminal His tag.

Shading Key
His-TEV-SpyCatcher-GS linker-SpyCatcher-GS linker-SpyCatcher

DNA
ATGTCGTACTACCATCACCATCACCATCACGATTACGATATCCCAACGACCGAA
AACCTGTATTTTCAGGGCGCCATGGGATCCGCCATGGTTGATACCTTATCAGGT
TTATCAAGTGAGCAAGGTCAGTCCGGTGATATGACAATTGAAGAAGATAGTGCT
ACCCATATTAAATTCTCAAAACGTGATGAGGACGGCAAAGAGTTAGCTGGTGCA
ACTATGGAGTTGCGTGATTCATCTGGTAAAACTATTAGTACATGGATTTCAGATG
GACAAGTGAAAGATTTCTACCTGTATCCAGGAAAATATACATTTGTCGAAACCG
CAGCACCAGACGGTTATGAGGTAGCAACTGCTATTACCTTTACAGTTAATGAGC
AAGGTCAGGTTACTGTAAATGGCAAAGCAACTAAAGGTGACGCTCATATTGGCG
GCTCCGGTGGTAGCAGATCCGCCATGGTTGATACCTTATCAGGTTTATCAAGTG
AGCAAGGTCAGTCCGGTGATATGACAATTGAAGAAGATAGTGCTACCCATATTA
AATTCTCAAAACGTGATGAGGACGGCAAAGAGTTAGCTGGTGCAACTATGGAGT
TGCGTGATTCATCTGGTAAAACTATTAGTACATGGATTTCAGATGGACAAGTGAA
AGATTTCTACCTGTATCCAGGAAAATATACATTTGTCGAAACCGCAGCACCAGA
CGGTTATGAGGTAGCAACTGCTATTACCTTTACAGTTAATGAGCAAGGTCAGGT
TACTGTAAATGGCAAAGCAACTAAAGGTGACGCTCATATTGGCGGCTCCGGTG
GTAGCAGATCTGCCATGGTTGATACCTTATCAGGTTTATCAAGTGAGCAAGGTC
AGTCCGGTGATATGACAATTGAAGAAGATAGTGCTACCCATATTAAATTCTCAAA
ACGTGATGAGGACGGCAAAGAGTTAGCTGGTGCAACTATGGAGTTGCGTGATT
CATCTGGTAAAACTATTAGTACATGGATTTCAGATGGACAAGTGAAAGATTTCTA
CCTGTATCCAGGAAAATATACATTTGTCGAAACCGCAGCACCAGACGGTTATGA
GGTAGCAACTGCTATTACCTTTACAGTTAATGAGCAAGGTCAGGTTACTGTAAAT
GGCAAAGCAACTAAAGGTGACGCTCATATTAGATCTTAG

Expression Product Sequence
MSYYHHHHHHDYDIPTTENLYFQGAMGSAMVDTLSGLSSEQGQSGDMTIEEDSAT
HIKFSKRDEDGKELAGATMELRDSSGKTISTWISDGQVKDFYLYPGKYTFVETAAP
DGYEVATAITFTVNEQGQVTVNGKATKGDAHIGGSGGSRSAMVDTLSGLSSEQGQ
SGDMTIEEDSATHIKFSKRDEDGKELAGATMELRDSSGKTISTWISDGQVKDFYLYP
GKYTFVETAAPDGYEVATAITFTVNEQGQVTVNGKATKGDAHIGGSGGSRSAMVD
TLSGLSSEQGQSGDMTIEEDSATHIKFSKRDEDGKELAGATMELRDSSGKTISTWIS
DGQVKDFYLYPGKYTFVETAAPDGYEVATAITFTVNEQGQVTVNGKATKGDAHIRS
*

SpyCatcher 4

**Name**	SpyCatcher GSx4 array	**Source**	5
**Resistance**	Ampicillin	**Total plasmid size (bp)**	6129
**Parent Vector**	pPROEX HTa	**Seq Primers**	M13_pUC +pBAD
**Benchling link**	https://benchling.com/s/seq-R6ZITqWrQ8LEwOXN7isx?m=slm-18jeUcdgKHXQBq1rpsEs		

Description
Four SpyCatcher units linked by GS flexible linker. Cleavable N terminal His tag.

Shading Key
His-TEV-SpyCatcher-GS linker-SpyCatcher-GS linker-SpyCatcher-GS linker-SpyCatcher

DNA

ATGTCGTACTACCATCACCATCACCATCACGATTACGATATCCCAACGACCGAA
AACCTGTATTTTCAGGGCGCCATGGGATCCGCCATGGTTGATACCTTATCAGGT
TTATCAAGTGAGCAAGGTCAGTCCGGTGATATGACAATTGAAGAAGATAGTGCT
ACCCATATTAAATTCTCAAAACGTGATGAGGACGGCAAAGAGTTAGCTGGTGCA
ACTATGGAGTTGCGTGATTCATCTGGTAAAACTATTAGTACATGGATTTCAGATG
GACAAGTGAAAGATTTCTACCTGTATCCAGGAAAATATACATTTGTCGAAACCG
CAGCACCAGACGGTTATGAGGTAGCAACTGCTATTACCTTTACAGTTAATGAGC
AAGGTCAGGTTACTGTAAATGGCAAAGCAACTAAAGGTGACGCTCATATTGGCG
GCTCCGGTGGTAGCAGATCCGCCATGGTTGATACCTTATCAGGTTTATCAAGTG
AGCAAGGTCAGTCCGGTGATATGACAATTGAAGAAGATAGTGCTACCCATATTA
AATTCTCAAAACGTGATGAGGACGGCAAAGAGTTAGCTGGTGCAACTATGGAGT
TGCGTGATTCATCTGGTAAAACTATTAGTACATGGATTTCAGATGGACAAGTGAA
AGATTTCTACCTGTATCCAGGAAAATATACATTTGTCGAAACCGCAGCACCAGA
CGGTTATGAGGTAGCAACTGCTATTACCTTTACAGTTAATGAGCAAGGTCAGGT
TACTGTAAATGGCAAAGCAACTAAAGGTGACGCTCATATTGGCGGCTCCGGTG
GTAGCAGATCTGCCATGGTTGATACCTTATCAGGTTTATCAAGTGAGCAAGGTC
AGTCCGGTGATATGACAATTGAAGAAGATAGTGCTACCCATATTAAATTCTCAAA
ACGTGATGAGGACGGCAAAGAGTTAGCTGGTGCAACTATGGAGTTGCGTGATT
CATCTGGTAAAACTATTAGTACATGGATTTCAGATGGACAAGTGAAAGATTTCTA
CCTGTATCCAGGAAAATATACATTTGTCGAAACCGCAGCACCAGACGGTTATGA
GGTAGCAACTGCTATTACCTTTACAGTTAATGAGCAAGGTCAGGTTACTGTAAAT
GGCAAAGCAACTAAAGGTGACGCTCATATTGGCGGCTCCGGTGGTAGCAGATC
TGCCATGGTTGATACCTTATCAGGTTTATCAAGTGAGCAAGGTCAGTCCGGTGA
TATGACAATTGAAGAAGATAGTGCTACCCATATTAAATTCTCAAAACGTGATGAG
GACGGCAAAGAGTTAGCTGGTGCAACTATGGAGTTGCGTGATTCATCTGGTAAA
ACTATTAGTACATGGATTTCAGATGGACAAGTGAAAGATTTCTACCTGTATCCAG
GAAAATATACATTTGTCGAAACCGCAGCACCAGACGGTTATGAGGTAGCAACTG
CTATTACCTTTACAGTTAATGAGCAAGGTCAGGTTACTGTAAATGGCAAAGCAA
CTAAAGGTGACGCTCATATTAGATCTTAG

Expression Product Sequence

MSYYHHHHHHDYDIPTTENLYFQGAMGSAMVDTLSGLSSEQGQSGDMTIEEDSAT
HIKFSKRDEDGKELAGATMELRDSSGKTISTWISDGQVKDFYLYPGKYTFVETAAP
DGYEVATAITFTVNEQGQVTVNGKATKGDAHIGGSGGSRSAMVDTLSGLSSEQGQ
SGDMTIEEDSATHIKFSKRDEDGKELAGATMELRDSSGKTISTWISDGQVKDFYLYP
GKYTFVETAAPDGYEVATAITFTVNEQGQVTVNGKATKGDAHIGGSGGSRSAMVD
TLSGLSSEQGQSGDMTIEEDSATHIKFSKRDEDGKELAGATMELRDSSGKTISTWIS
DGQVKDFYLYPGKYTFVETAAPDGYEVATAITFTVNEQGQVTVNGKATKGDAHIGG
SGGSRSAMVDTLSGLSSEQGQSGDMTIEEDSATHIKFSKRDEDGKELAGATMELR
DSSGKTISTWISDGQVKDFYLYPGKYTFVETAAPDGYEVATAITFTVNEQGQVTVN
GKATKGDAHIRS*

3. Strains used in this study

**Strains**	**Relevant Characteristics**	**Source**
E. coli		
BL21 Gold(DE3)		Professor Lynne Regan's lab stock (University of Edinburgh)
**Plasmids**		
SpyTag-SasG-SpyTag	T7 promoter-operator, N-terminal His tag, Amp^r, plasmid for expression of ST-SasG-ST	1
SpyTag-SasGlong-SpyTag	T7 promoter-operator, N-terminal His tag, Amp^r, plasmid for expression of ST-SasGlong-ST	1
SpyCatcher – GS 3 Array	T7 promoter-operator, N-terminal His tag, Amp^r, plasmid for expression of SC3	1
SpyCatcher – GS 4 Array	T7 promoter-operator, N-terminal His tag, Amp^r, plasmid for expression of SC4	1

References

1. A. Edelstein, Amodaj, N., Hoover, K., Vale, R., Stuurman, N., *Curr. Protoc. Mol. Biol.*, 2010, **92**, 14.20.11-14.20.17.
2. W. Mulyasasmita, Lee, J.S., Heilshorn, S.C., *Biomacromolecules*, 2011, **12**, 3406–3411.
3. T. Savin, Doyle, P.S., *Biophysical Journal*, 2005, **88**, 623-638.
4. J. C. Crocker, Grier, D.G, *J. Colloid Interface Sci.*, 1996, **179**, 298.
5. D. Williams, *Yale University* 2018.
